# Ecological momentary assessment of the stress-recovery process through technology mHealth in sports: a scoping review

**DOI:** 10.1186/s13102-026-01634-8

**Published:** 2026-03-07

**Authors:** Joel Guillen Cots, Lluis Capdevila, Josep-Maria Losilla, Eva Parrado

**Affiliations:** 1 Laboratory of Sport Psychology, Department of Basic Psychology, Universitat Autònoma de Barcelona, Bellaterra, Spain; 2 Sport Research Institute, Universitat Autònoma de Barcelona, Bellaterra, Spain; 3 Department of Psychobiology and Methodology of Health Sciences, Faculty of Psychology, Autonomous University of Barcelona, Bellaterra, Barcelona, Spain

**Keywords:** Athlete monitoring, EMA, mobile health, fatigue, well-being, training load

## Abstract

**Background:**

This scoping review aimed to identify how Ecological Momentary Assessment methodologies using mobile health technologies are applied to monitor training load and the stress-recovery process in athletes. The review focused on mapping the technologies and methodologies employed, examining their use in team and individual sports, and exploring the relationships between external and internal load measures, as well as the extent to which subjective and objective indicators are integrated within monitoring systems.

**Method:**

Following PRISMA-ScR guidelines, a systematic search across four databases identified 71 studies published between 2012 and 2025 that met eligibility criteria.

**Results:**

Half of studies focused on elite or international athletes (50.68%), with a predominance of male participants (63.4%). Team sports represented the vast majority (87.3%), particularly soccer (42.5%) and basketball (15.1%). A wide range of technologies was reported, from advanced wearable systems to smartphone applications, with external load commonly tracked via GPS and accelerometry, and internal load through heart rate indices, Training Impulse, Rating of Perceived Exertion, and wellness questionnaires. However, only seven studies directly compared subjective and objective measures of internal load, revealing partial convergence and underscoring the value of multimodal monitoring strategies.

**Conclusions:**

Ecological Momentary Assessment-based mobile health systems provide valuable insights into athlete load and recovery, but important gaps remain in individual sports, female representation, and recovery-related behaviors. Future research should prioritize methodological transparency, broader inclusion of populations, and the adoption of emerging non-invasive biosensing technologies to achieve a more holistic and ecologically valid model of athlete monitoring.

**Supplementary Information:**

The online version contains supplementary material available at 10.1186/s13102-026-01634-8.

## Background

During physical activity, athletes face situations that can affect their performance. These situations may be related to the demands of the sport itself or to external issues, causing physical or psychological fatigue. Conceptually, fatigue can be understood both as a progressive decrement in performance over time (fatigability) and as the subjective experience of needing rest or perceiving a discrepancy between the effort invested and the performance achieved [1]. The inability to sustain athletic performance, together with inadequate rest and recovery, may constitute a significant source of stress for athletes. To preserve well-being and sustain optimal performance, athletes must therefore prioritize recovery. Effective management of the stress–recovery process can only be achieved by considering all relevant factors, including training characteristics, sleep, nutrition, social environment, and health status, among others, and should consequently be approached as holistically as possible [2–4].

Within sport science, a central objective is to monitor and interpret athletes’ status to mitigate injury, illness, and non-functional overreaching [2, 5] and to ensure day-to-day readiness [6]. The work performed in training and competition is typically summarized as training load [7] and comprises both external and internal components [8]. Measuring the external load gives information about the work done by the athletes, while measuring the internal load relates to knowing the stress that the body has been subjected to [9]. There are several parameters used to evaluate both internal and external load. Parameters of time, distance, power output or composite methods derived from acceleration (Player Load) correspond to external load [10, 11], while blood lactate, oxygen uptake, heart rate indices, rating of perceived exertion (RPE) or wellness questionnaires correspond to internal load, among others [9, 10]. Both measures are important; however, the key to determining whether an athlete is fatigued lies in the relationship between external and internal load [6] and in the integration of these measures within a holistic framework of fatigue assessment, thereby enabling a more individualized training process [12]. For example, high internal load levels when the external load is low, may indicate a lack of adaptation to training. Moreover, athletes can be in a state of underrecovery not only due to high stress but also as a result of insufficient recovery [3]. To restore the resources that have been invested, athletes need to engage in recovery strategies, which may be either physical or psychological. Periodization and monitoring of recovery is important for optimal athlete performance [13], as athletes spend more time recovering than training itself [14].

Technological advancements have enabled the creation of comprehensive monitoring systems that offer detailed insights into an athlete’s performance and health [15, 16]. Mobile health (mHealth) is the use of mobile and wireless technologies (e.g., smartphones, apps, and wearable sensors) to deliver health-related services and to collect health data in real time [17]. The combination between these devices and advanced analytics software has become a cornerstone of contemporary training programs. These tools provide real-time data within athletes’ natural environments and thereby facilitate Ecological Momentary Assessment (EMA). EMA refers to the repeated, real-time assessment of individuals’ behaviors, experiences, and physiological states in their everyday contexts, reducing recall bias and increasing ecological validity [18]. This methodological approach enables coaches and sports scientists to obtain rich, timely information to guide decision-making regarding athletes’ status [11, 19]. This is especially important in athlete monitoring, as EMA is well suited to capturing the dynamic nature of stress and recovery, which can fluctuate across days and within the same day in response to training, competition, and contextual demands [3]. Furthermore, with the increasing reliance on objective metrics, recent perspectives emphasize the need to integrate subjective monitoring to achieve a more holistic understanding of athletes’ states [20]. Nevertheless, sports staff generally prefer assessment tools that are simple, easy to use, time-efficient, non-invasive, and non-fatiguing for athletes [5, 21]. Given the wide variety of training load measures and monitoring systems available, there is a need-to-know not only which measures and technologies are the most used but also in what context, for which type of sport, and which might be the most suitable.

Previous studies have addressed this need by conducting systematic reviews to classify the measures used to examine the state of athletes [22–25] and the technological devices employed for monitoring [26–28]. In their meta-analysis, McLaren et al. [29] reported associations among wearable-derived variables, including links between heart-rate-based training impulse (TRIMP) and total distance covered. More recently, Helwig et al. [30] summarized wearable-based relationships between external and internal indicators, highlighting the frequently studied pairing of session RPE (sRPE) with accelerometry-derived load. However, both reviews primarily focused on team sports athletes, overlooking individual sports athletes and considered invasive technology for the monitoring of the internal parameters. Notably, female athletes remain underrepresented in the athlete-monitoring literature, potentially limiting the generalizability of existing findings [31]. Accordingly, applying monitoring frameworks derived primarily from male athletes may be problematic, as sex-specific physiology and hormone fluctuations can influence commonly used internal-load and recovery markers such as HRV [32].

For this reason, the objective of this scoping review is (a) to identify EMA methodologies through mHealth technology to monitor the training load or the stress-recovery process in athletes, (b) to classify the methodologies and technology used according to individual or team sports, (c) to describe the relationship between external parameters (distance, accelerations, decelerations, etc.) and internal parameters (physiological, cardiovascular, neuromuscular, subjective, etc.), and (d) to analyze the relationship between subjective and objective parameters of the internal measures.

## Method

The study was carried out in conformity with the PRISMA Extension for Scoping Reviews (PRISMA-ScR) recommendations [33]. The protocol for this scoping review was previously registered at the International Platform of Registered Systematic Review and Meta-Analysis Protocols (INPLASY) with the registration number: INPLASY202330089 [34]. In accordance with the principles of open science, all datasets and supplementary information referenced in the following sections are publicly available in the CORA repository and can be accessed at 10.34810/data2613.

### Search strategy

The search for the primary studies was carried through four databases: APA PsycInfo by ProQuest, PubMed by US National Center for Biotechnology Information, Web of Science Core Collection by Clarivate, and SPORTDiscus by EBSCOHost.

The search strategy followed the Peer Review of Electronic Search Strategies (PRESS) [35] and PRISMA for Searching (PRISMA-S) guidelines [36], and consisted of three groups of search terms referring to: (a) sports and physical activity, (b) wireless/wearable technologies and mobile applications, and (c) training load or stress-recovery state. We also added a fourth group of terms preceded by the Boolean operator NOT to improve the specificity of the search strategy and exclude injured athletes.

The generic search expression used was: (“sport$” OR “athlet*” OR “exercis*” OR “physical activit*”) AND (“mobile health” OR “mHealth” OR “eHealth” OR “e-Health” OR “wearable$” OR “wireless technolog*” OR “microsensor$” OR “microtechnolog*” OR “GPS” OR ”accelerometer” OR “training monitoring” OR “mobile app*” OR “smartphone app*”) AND (“stress” OR “fatigue” OR “load” OR “workload” OR “overtraining” OR “underrecovery” OR “staleness” OR “overreaching” OR “rest” OR “recovery” OR “exhaustion” OR “tiredness” OR “readiness”) NOT (“Injur*”). The specific syntax adapted to each of the bibliographic databases consulted is available in the supplementary file “Database-specific search syntax”.

The search was performed on 6 April 2025 and was restricted to publications from 2012 to 2025. The year 2012 was selected because it marks a turning point in smartphone use (e.g., smartphone adoption in Europe reached approximately 47.6% [37] and in the systematic use of mobile devices to promote physical activity [38]. Only articles published in English or Spanish were included.

### Study selection

Eligible studies involved athletes aged 13–65 (youth, amateur, or high-performance) in team or individual sports, using EMA via mHealth to monitor training load or stress–recovery, with experimental, single-case, cohort, or cross-sectional designs. We excluded studies on injured athletes; validation/reliability papers without monitoring; one-off lab/field tests without longitudinal monitoring; and those measuring only internal or only external load or lacking their relationship. Full criteria are detailed in the previously published protocol [34].

References identified by the search strategy were entered into Mendeley bibliographic software, and duplicates were removed by one reviewer (JG). Two reviewers (JG & EP) independently screened the articles in two phases. In the first phase, the inclusion and exclusion criteria were applied to all titles and abstracts. Articles meeting the inclusion criteria were chosen, and in cases where decisions could not be reached based solely on the title and abstract, the complete article was retrieved. In the second phase, the full-text inclusion and exclusion criteria was assessed. A third author (LC) solved discrepancies during the process. Cohen’s kappa coefficient was calculated to measure the agreement between the reviewers during the reviewing process.

### Data management/data extraction

A data extraction protocol was designed for the selected studies (supplementary file “Data extraction”). The extracted information included:

- General information: title, author, year of publication and journal.
- Research design.
- Characteristics of the target population of the study: number of participants, age, gender, sport level and sport type.
- The monitoring process: technology, stress-recovery variables (based on Bourdon et al. [10], periodicity and the main results.

Since our exploratory review was designed to be exploratory and descriptive, which differs fundamentally from the analytical and evidence-based approach of systematic reviews, no formal assessment of the quality of the included studies was performed [39].

### Data analysis

A narrative synthesis was performed to analyze and present the results. Information extracted from the included studies was organized in tables and complemented by graphical representations to facilitate the identification of patterns and trends. The analysis of the results was carried out taking into account the variability that could be related to the following moderating variables: year of publication, gender, age, analysis approach and geographical origin of the study, type of sport and whether it was classified as an individual or team sport, and the types of portable devices used to monitor the internal or external load variables in each study.

## Results

### Study selection

**Fig. 1:**
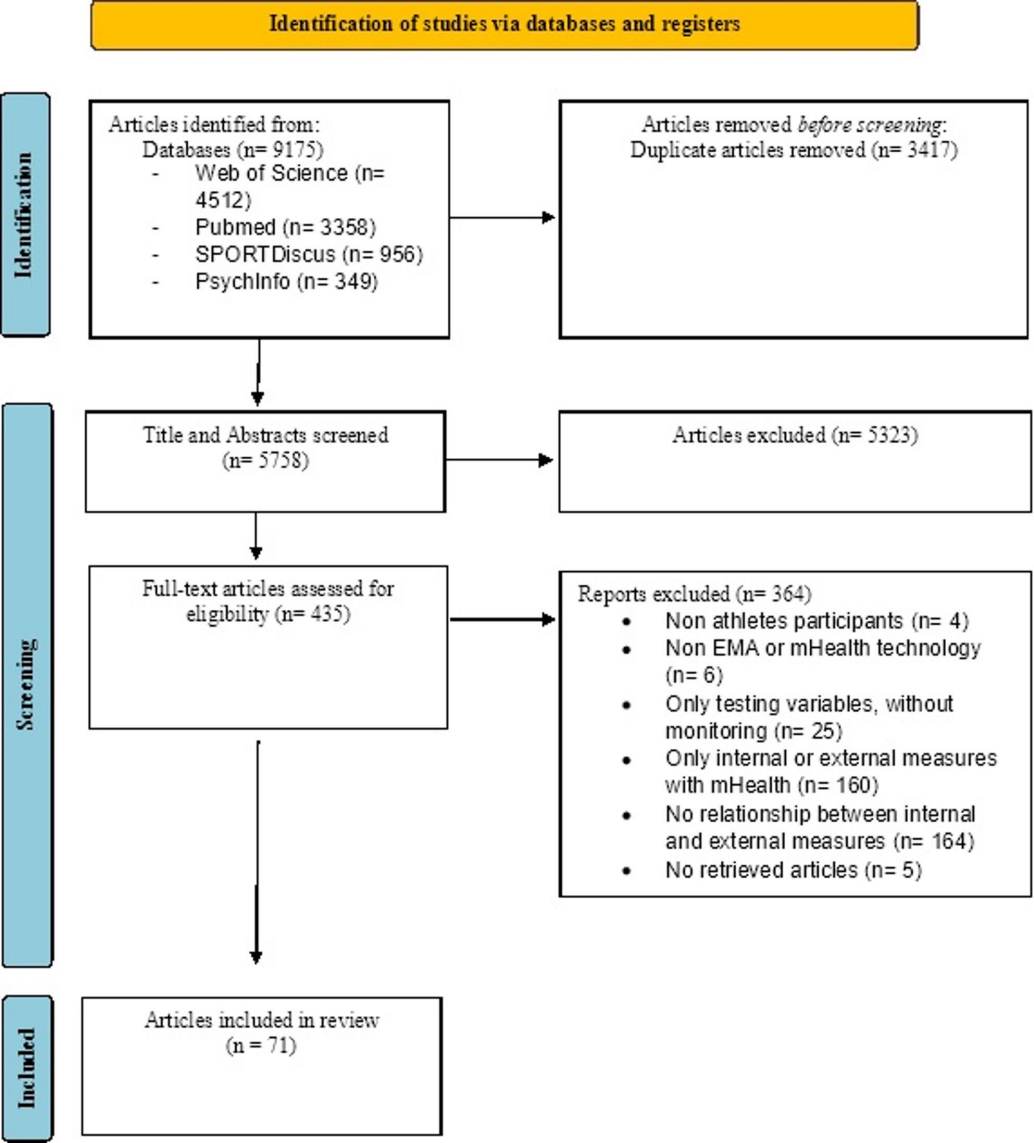
PRISMA flowchart of the scientific literature search and the selection of studies

The search identified a total of 9175 articles across the four databases. After eliminating duplicate studies and screening by title and abstract, 435 studies were selected for review based on their full text. Ultimately, 71 studies met the eligibility criteria and were included in the review (see Fig. 1). A very good agreement between the reviewers was shown, as the Cohen’s kappa coefficient was 1 in the first phase and 0.836 (I.C 95%: 0.746–0.925) in the second phase.

### Characteristics of the selected studies

**Fig. 2:**
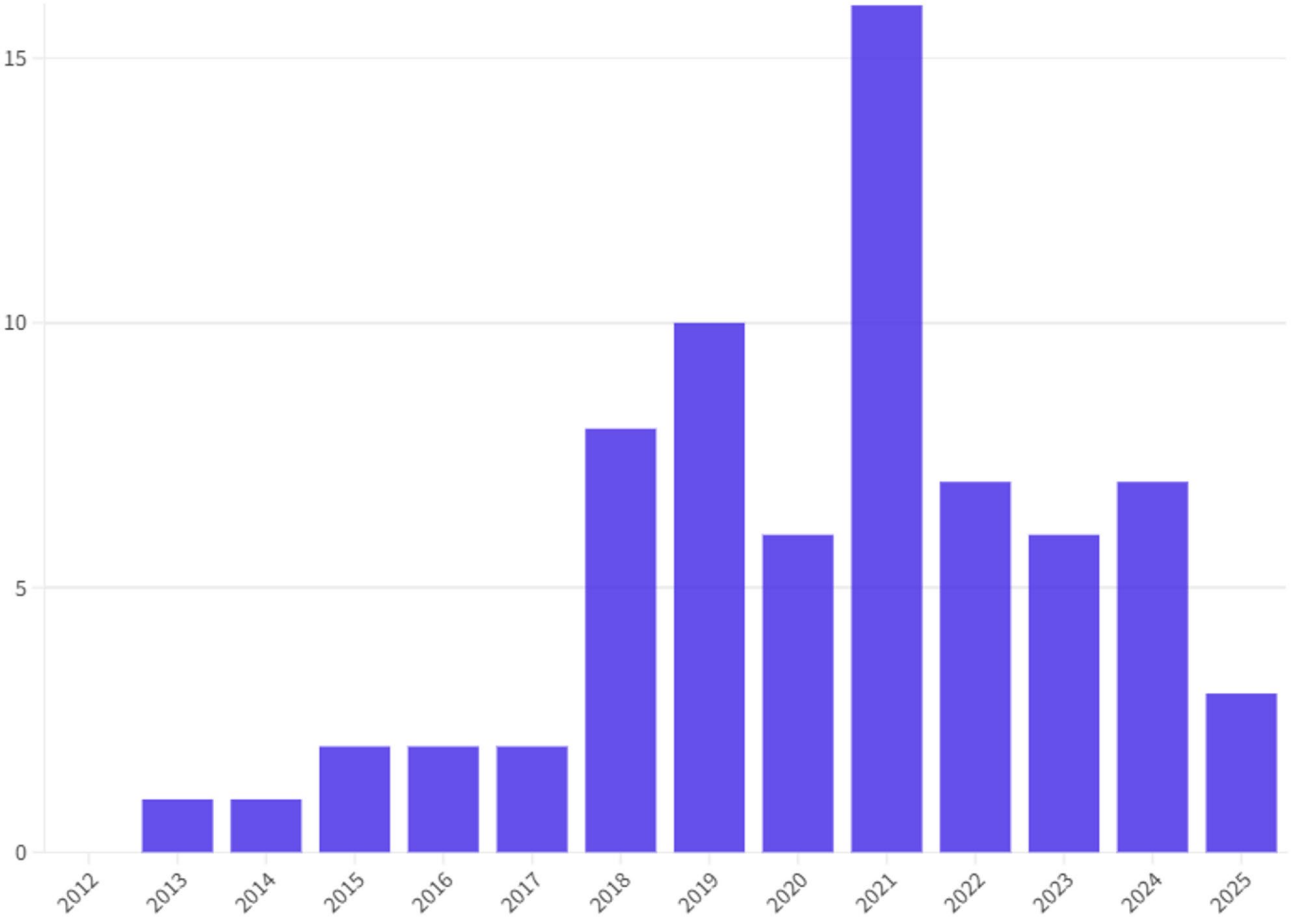
Number of included studies by publication year since 2012

Figure 2 shows the number of papers published every year since 2012. The years with more publications of studies included were 2021 (*n* = 16) and 2019 (*n* = 10).

Regarding participant characteristics, most studies included only male athletes (*n* = 1120), and only 21 (29.57%) focused on female athletes (*n* = 323). Additionally, four studies included mixed-gender participants. As for the number of participants, most studies included between 11 and 30, and only 11 studies (15.49%) had more than 30 participants. Regarding age distribution, according to the available data (with seven articles not providing this information), the reported mean ages range from 14.14 to 38.85 (with corresponding standard deviations ranging from 0.40 to 8.88). Regarding competitive level [40], approximately half of the athletes were classified as elite or international, most of the remaining participants competed at a highly trained national level, and only a small proportion were considered trained or developmental. As for analytical approaches, most studies employed conventional methods (descriptive, correlational, or regression analyses), and only a quarter used advanced techniques such as principal component analysis, machine learning, or mixed models. In terms of location, the United States and Australia were the most represented countries, followed by Spain and Portugal, with the remaining studies distributed across Europe, Africa, and Asia (see Table 1). The included studies generally reported evidence of device validity and/or reliability for objective measures of external load and internal load, whereas greater heterogeneity was observed in the assessment of subjective internal load. Reporting of missing data also varied across studies: some attributed missingness to injury or playing role/position, and several did not report reasons for missing data. Importantly, all studies described the procedures followed during data collection, with protocols tailored to the specific sport context, the monitoring technologies employed, and the variables assessed.

**Table 1:** Frequency distribution of general characteristics

Variable	Category	Frequency	
		*n*	%
Gender	Female	21	29.57
	Male	45	63.38
	Mixed	4	5.63
	Not specified	1	1.40
Participants	1–10	17	23.94
	11–30	42	59.15
	> 30	11	15.49
	Not specified	1	1.41
Level*	Elite/International	37	50.68
	Highly trained/National	32	43.83
	Trained/Developmental	4	5.47
Analysis†	Advanced	19	26.76
	Conventional	52	73.24
Country	United States of America	15	21.12
	Australia	13	18.30
	Spain	9	12.67
	Portugal	6	8.45
	France	2	2.81
	Lithuania	2	2.81
	Sweden	2	2.81
	Canada	2	2.81
	United Kingdom	2	2.81
	Italy	2	2.81
	Ireland	2	2.81
	South Africa	2	2.81
	Other countries‡	12	16.90
Sport§	Soccer	31	42.46
	Basketball	11	15.06
	Rugby	4	5.47
	Hockey	3	4.10
	American Football	3	4.10
	Badminton	2	2.73
	Gaelic football	2	2.73
	Ice Hockey	2	2.73
	Lacrosse	2	2.73
	Other sports¶	13	17.80

* Level classification based on McKay et al. [40] framework

† Analysis advanced includes principal component analysis, machine learning and mixed models. Conventional includes descriptive, correlational and regression analyses

‡ Other countries (*n* = 1): Chile, Malaysia, Belgium, Germany, Russia, Brazil, Norway, Denmark, Qatar, Iran, Singapore, Mexico

§ The total number of sports included is 73, since Campbell et al. [41] studies three sports

¶ Other sports (*n* = 1): Surf, Cycling, Handball, Cross-Country, Squash, Netball, Volleyball, Beach Volleyball, Trail-Running, Cricket, Tennis, Biathlon, Waterpolo

### Sports participation

**Fig. 3:**
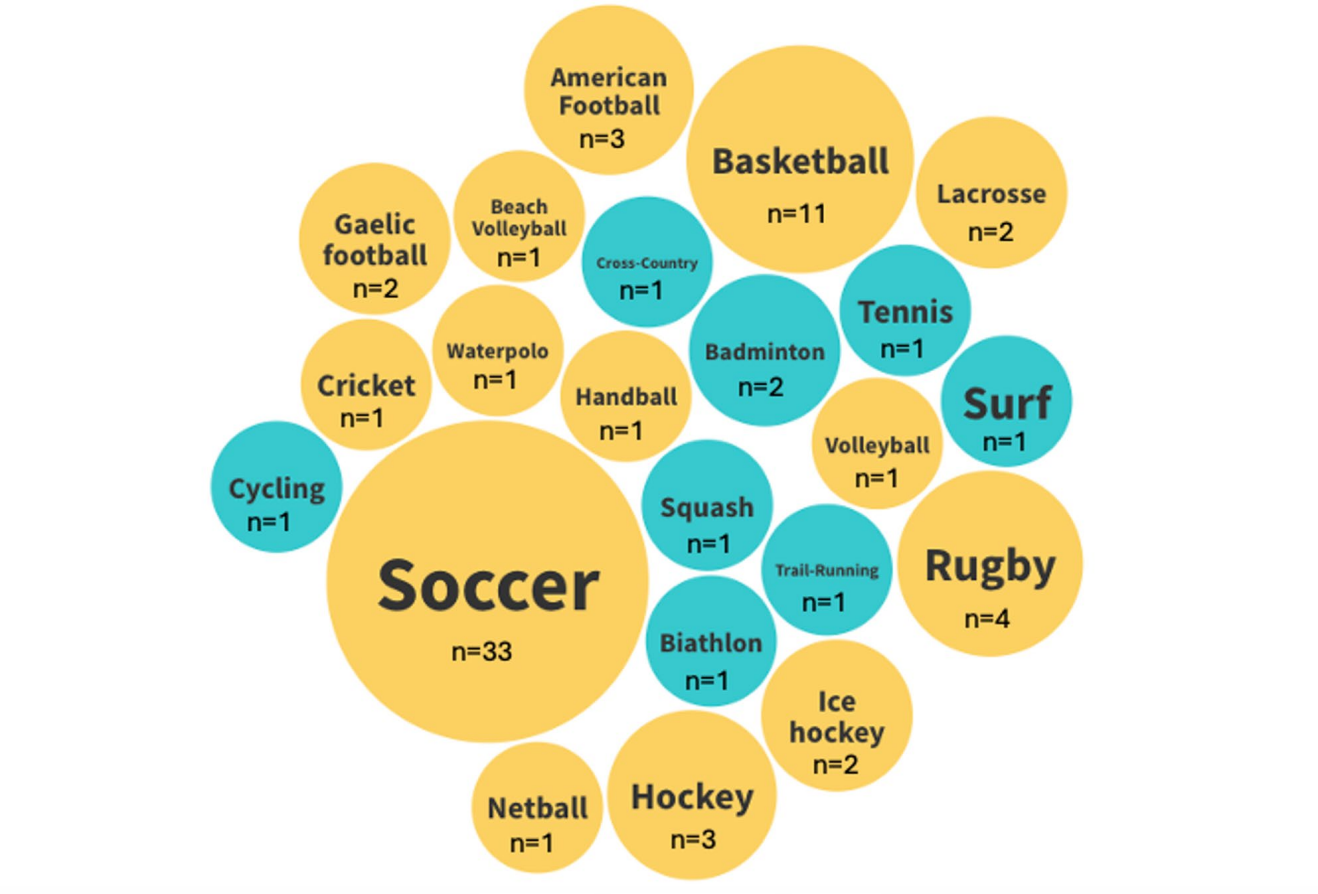
Sports and type of sports (individual - team) of the included studies. Individual sports in blue color and team sports in yellow

In relation to the sports included in the studies, soccer was the most present (*n* = 31, 42.46%), followed by basketball (*n* = 11, 15.06%) and rugby (*n* = 4, 5.47%). Figure 3 displays all the sports included, split by the type of sport, individual (blue, *n* = 9, 12.67%) or team (yellow, *n* = 62, 87.32%) sports.

The monitoring process of the included studies are summarized in Table 2, involving the training load measures employed and the duration of data collection. Three studies (4.22%) monitored athletes for less than one week, 14 studies (19.71%) for periods between two weeks and one month, and 16 studies (22.53%) for one to three months. Half of the studies (*n* = 36, 50.70%) collected data over periods longer than three months, whereas two studies did not report the duration of monitoring.

**Table 2:** Checklist of the monitoring process of the included studies

Study		Sports	Monitoring process																		
			Training load measures†																		
			Internal						External									Duration			
			Objective			Subjective															
			TRIMP	HRi	OA	RPE	sRPE	Wellness	Distance	PL	Speed	Acc/Dec	MRC	Time	PO	MP	Ac	<1w.	<1m.	1-3m.	>3m.
Abdullahi et al. (2019) [42]		Badminton		X					X	X				X							X
Askow et al. (2021) [43]		Soccer	X	X		X	X			X	X			X			X				X
Bellinger et al. (2020) [44]		Soccer						X	X	X	X										X
Bisschoff et al. (2016) [45]		Badminton		X					X	X		X	X	X							X
Campbell et al. (2021) [41]	Cricket, Rugby & Soccer				X		X	X		X										X	
Chrismas et al. (2019) [46]	Soccer		X					X		X					X					X	
Clemente (2018) [47]	Soccer		X					X	X	X								X			
Clements et al. (2025) [48]	Soccer						X	X		X	X									X	
Conte et al. (2021)[49]	Basketball		X		X	X	X		X									X			
Conte et al. (2021) [50]	Rugby						X	X		X	X							X			
Costa et al. (2019) [51]	Soccer		X		X	X		X		X							X				
Croteau et al. (2023) [52]	Water polo				X	X			X			X								X	
Crouch et al. (2021) [53]	Lacrosse	X			X		X	X	X											X	
Cullen et al. (2021) [54]	Soccer				X		X	X	X	X	X				X					X	
de Dios-Alvarez et al. (2023)[55]	Soccer				X		X	X	X	X	X				X					X	
Delaney et al. (2018)[56]	Rugby	X						X	X					X					X		
Douchet et al. (2021) [57]	Soccer		X		X	X		X			X	X						X			
Draper et al. (2021) [58]	Soccer	X			X	X	X	X	X	X			X							X	
Ellis et al. (2021) [59]	Soccer	X						X	X	X								X			
Evans et al. (2022)[60]	Soccer						X	X		X	X									X	
Fernandez et al. (2021) [61]	Hockey				X	X		X	X	X	X									X	
Fields et al. (2021) [62]	Soccer		X					X	X	X								X			
Fox et al. (2020)[63]	Basketball		X						X		X	X								X	
Fox et al. (2018) [64]	Basketball		X					X	X										X		
Gielen et al. (2022) [65]	Volleyball		X													X	O				
Gonzalez-Fimbres et al. (2019) [66]	Hockey	X							X											X	
Gónzalez-García & Romero-Moraleda (2024) [67]	Soccer						X	X	X	X							X				
Govus et al. (2018) [68]	American football						X		X										X		
Grace et al. (2023) [69]	Lacrosse		X				X	X			X	X								X	
Heishman et al. (2018) [70]	Basketball	X	X						X										X		
Henderson et al. (2019) [71]	Rugby						X	X		X			X							X	
Ihsan et al. (2017) [72]	Hockey				X		X	X		X	X							X			
James et al. (2021) [73]	Squash	X							X		X			X				X			
Javaloyes et al. (2021) [74]	Cycling		X						X										X		
Kårström et al. (2024) [75]	Biathlon	X														X			X		
Kniubaite et al. (2019) [76]	Handball		X						X											X	
Lacome et al. (2018) [77]	Soccer		X					X	X	X										X	
Lempke et al. (2022) [78]	Cross-Country				X		X									X			X		
Long et al. (2024) [79]	Soccer				X		X	X	X				X							X	
Lopez-Laval et al. (2022) [80]	Basketball	X	X					X								X			X		
Mara et al. (2015) [81]	Soccer						X	X		X	X									X	
Matos et al. (2019) [82]	Trail running				X		X	X					X					X			
Peterson & Quiggle (2017) [83]	Basketball			X					X							X				X	
Pexa et al. (2023) [84]	Soccer		X				X	X		X	X									X	
Power et al. (2024) [85]	Basketball		X						X							X				X	
Rabbani et al. (2019) [86]	Soccer	X	X					X	X	X									X		
Rago et al. (2022) [87]	Hockey		X								X							X			
Rago et al. (2025) [88]	Hockey	X	X								X							X			
Rahilly et al. (2023)[89]	Soccer				X	X	X	X		X									X		
Reina et al. (2019) [90]	Basketball		X						X											X	
Reinhardt et al. (2020) [91]	Soccer		X							X	X									X	
Renaghan et al. (2024) [92]	Soccer		X						X											X	
Sánchez-Sánchez et al. (2021) [93]	Soccer		X					X			X	X							X		
Sansone et al. (2022)[94]	Basketball		X		X	X			X										X		
Sansone et al. (2025) [95]	Basketball		X						X							X		X			
Scanlan et al. (2014) [96]	Basketball	X	X						X										X		
Scott et al. (2013) [97]	Soccer	X						X	X	X										X	
Scott & Lovell (2018) [98]	Soccer	X			X		X			X								X			
Silva et al. (2021) [99]	Surf		X		X	X		X		X			X						X		
Silva et al. (2018) [100]	Soccer	X	X							X	X							X			
Simpson et al. (2020) [101]	Netball		X						X		X	X								X	
Sobolewski (2020) [102]	American football	X	X		X	X		X		X	X	X							X		
Suarez-Arrones et al. (2015) [103]	Soccer		X							X										X	
Teixeira et al. (2023) [104]	Soccer	X	X					X	X	X	X				X				X		
Tendero-Ortiz et al. (2024) [105]	Tennis		X					X	X	X	X		X				X				
Tometz et al. (2022) [106]	Beach volleyball	X			X	X		X												X	
Torreno et al. (2016) [107]	Soccer		X					X		X										X	
Villaseca-Vicuña et al. (2023) [108]	Soccer				X		X	X	X	X		X					O				
Wellman et al. (2019) [109]	American football						X	X	X		X									X	
Wiig et al. (2020) [110]	Soccer				X	X		X	X											X	
Zafar et al. (2024) [111]	Soccer		X							X										X	

†Based on Bourdon et al. [10]. X: Indicates that the variable appears in the study, or the duration of data collection. O: Indicates that the duration of the data collection is not reported

*TRIMP* Training Impulse, *HRi* Heart rate indices, *OA* Other assessments, *RPE* Rating of Perceived Exertion, *sRPE* session Rating of Perceived Exertion, *PL* Player Load, *Acc/Dec* Acceleration/Deceleration, *MRC* Movement repetition counts, *PO* Power Output, *MP* Metabolic Power, *Ac* Accelerometry, *w* week, *m*. months

### External and internal load variables and technologies

**Fig. 4:**
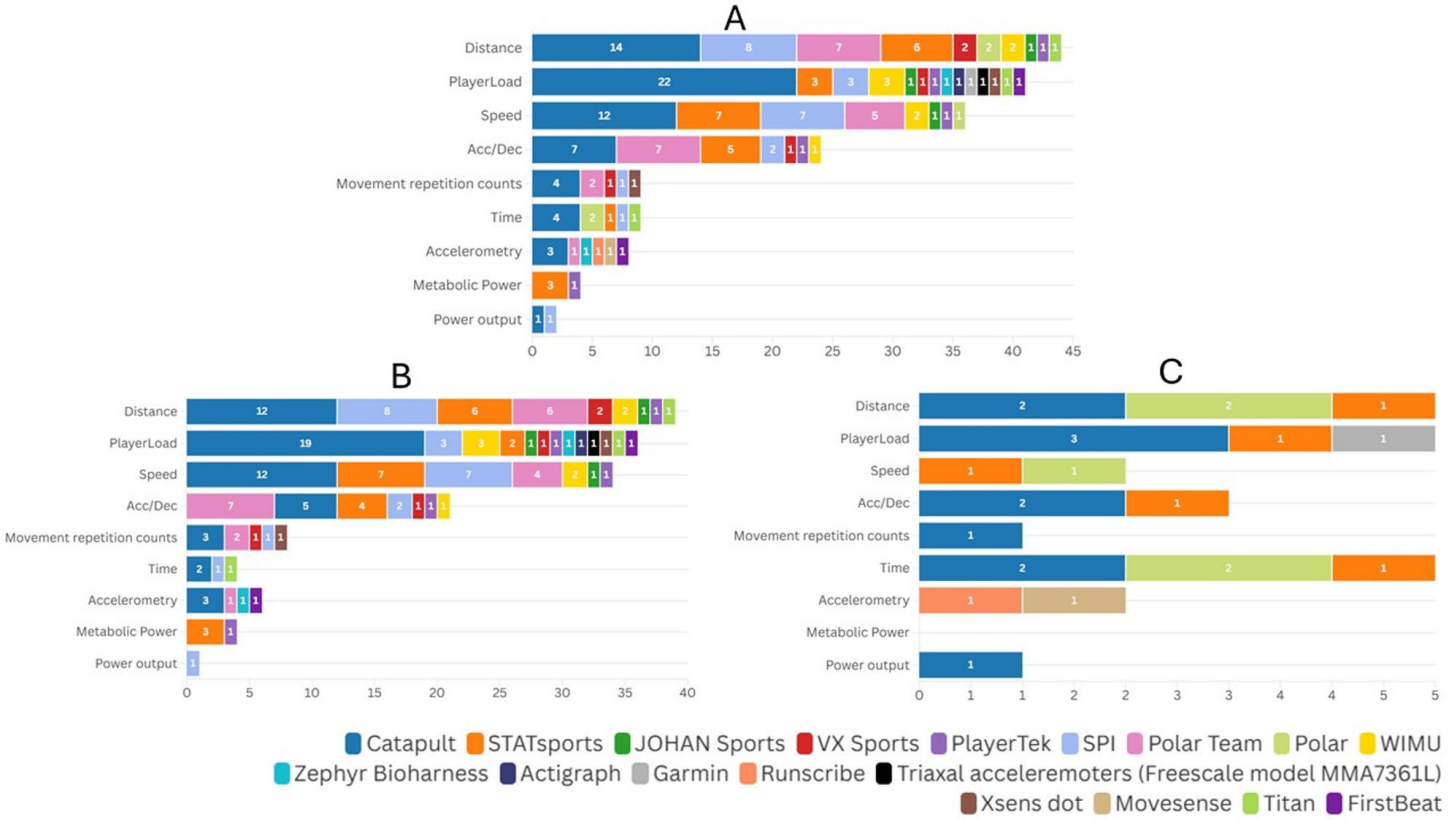
External load variables and technology trademark used in the included studies. **A** shows total values across all included studies, **B** shows team-sport studies, and **C** shows individual-sport studies. n: indicates the number of occurrences across all studies, not the number of studies. Acc/Dec: Acceleration/Deceleration. Example measurement units reported across studies include: distance (meters), Player Load (arbitrary units), speed (meters per second or kilometers per hour), accelerations/decelerations (meters per second squared), movement repetition counts (e.g., changes of direction, jumps), time (seconds or minutes), accelerometry (x-, y-, and z-axis g-force), metabolic power (watts per kilogram), and power output (joules)

The most frequently reported external load variables were distance (*n* = 44, 24.86%), Player Load (*n* = 41, 23.16%), speed (*n* = 36, 20.34%), and acceleration/deceleration (*n* = 24, 13.56%). These metrics were commonly monitored using branded systems such as Catapult (*n* = 26, 36.62% of studies, e.g., [36, 56, 78]), Polar Team (*n* = 9, 12.68%, e.g., [73, 80, 81]), SPI (*n* = 9, 12.68%, e.g., [49, 96, 100]), STATSports (*n* = 8, 11.27%, e.g., [39, 41, 97]). Other technologies such as WIMU [61, 67, 90], Zephyr Bioharness [65, 94] and PlayerTek [55] were used less frequently. Regarding the integration of technology trademarks and external load variables (Fig. 4A), distance was mainly assessed with Catapult (*n* = 14) and SPI (*n* = 8); Player Load almost exclusively with Catapult (*n* = 22); speed with Catapult (*n* = 12) and STATSports or SPI (*n* = 7); and acceleration/deceleration with Catapult or Polar Team (*n* = 7). When stratified by sport type, Player Load (*n* = 19) and distance and speed (both *n* = 12) were the most frequently reported variables in team sports (Fig. 4B), whereas in individual sports (Fig. 4C) time, distance, and Player Load (each *n* = 5) were reported most frequently.

**Fig. 5:**
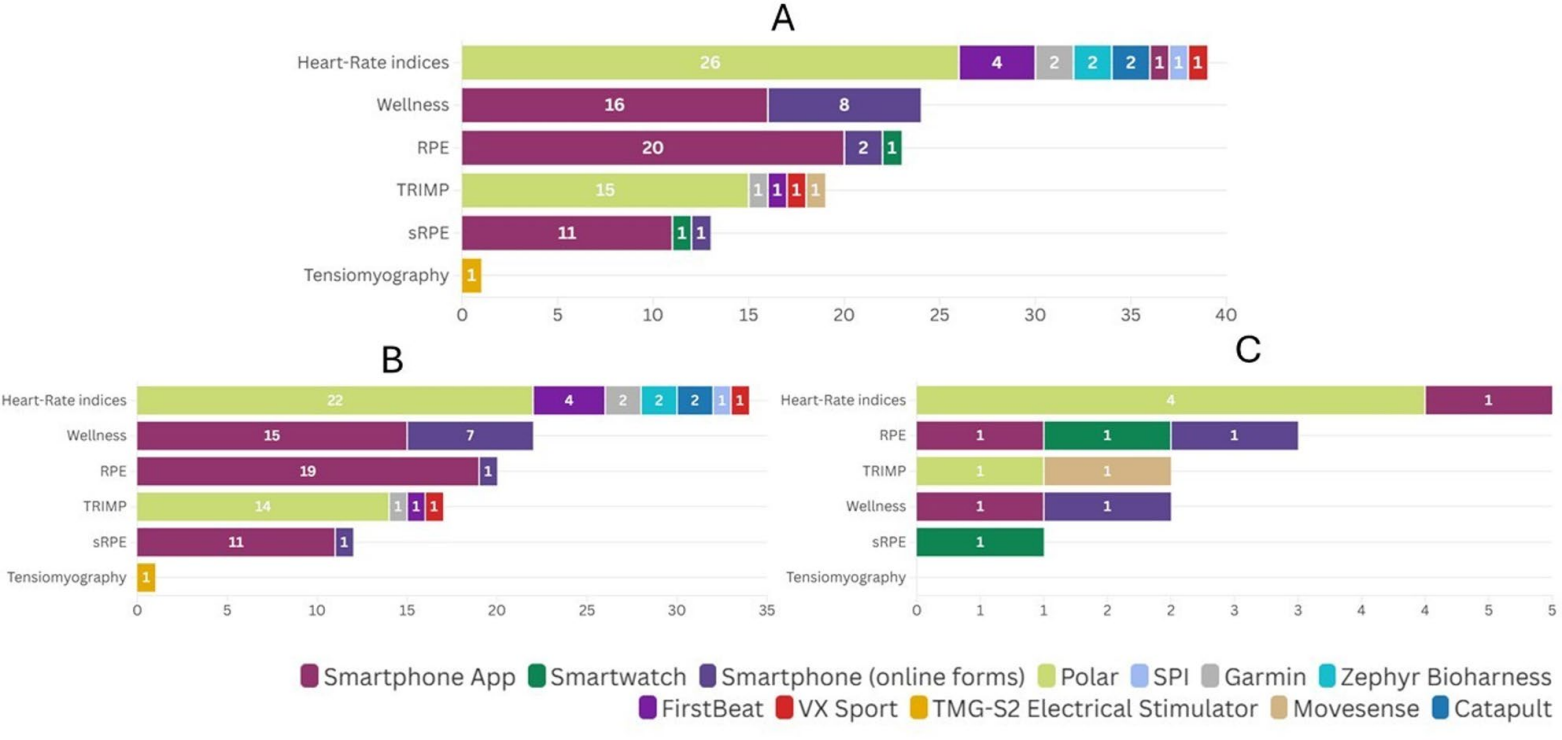
Internal load variables and technology trademarks used in the included studies. **A** shows total values across all included studies, **B** shows team-sport studies, and **C** shows individual-sport studies. n: indicates the number of occurrences across all studies, not the number of studies. TRIMP: Training Impulse. HR indices: Heart rate indices. RPE: Rating of Perceived Exertion. sRPE: session Rating of Perceived Exertion. Example measurement units reported across studies include: RPE (0–10 Likert scale), sRPE (arbitrary units; calculated as RPE × session duration in minutes), wellness (arbitrary units from Likert scales; e.g., 1–5 or 1–7), heart-rate indices (beats per minute for HRmean/HRmax; percentage of HRmax; and milliseconds for HRV metrics such as RMSSD or SDNN), TRIMP (arbitrary units), and tensiomyography (milliseconds for contraction time)

As shown in Fig. 5, the most used objective internal load variables included heart rate indices (*n* = 39, 32.77%) and TRIMP scores (*n* = 19, 15.96%). The most commonly used technology trademarks to collect objective measures were Polar (*n* = 34, 69.39% of studies, e.g., [35, 92, 95, 99]) and FirstBeat (*n* = 4, 8.16%, e.g., [44, 63, 86, 88]).The most frequently reported subjective internal load variables were Wellness (*n* = 24, 20.17%), RPE (*n* = 23, 19.33%), and sRPE (*n* = 13, 10.42%). Wellness measures commonly included self-reported perceptions of fatigue, stress, muscle soreness, and sleep quality, and were assessed using different Likert scales (e.g., 1–5, 0–10, or 1–7), whereas RPE was consistently reported using a 0–10 Likert scale. These were mostly collected through smartphone apps (*n* = 25, 73.53% of studies, e.g., [36, 46, 51]) like TeamBuildr or online forms (*n* = 8, 23.53%, e.g., [60, 74, 75]). Regarding the integration between technology trademarks and variables (Fig. 5A), Polar was mainly used to capture heart rate indices (*n* = 26) and TRIMP (*n* = 15). Smartphone apps were most frequently used for RPE (*n* = 20), sRPE (*n* = 11), and Wellness (*n* = 16). When analyzed by sport type, heart rate indices were the most frequently reported variables in both team (Fig. 5B) and individual sports (Fig. 5C).

### Associations between external and internal load

Moderate to very strong correlations between external and internal load were reported in 28 studies, with representative examples including several investigations (e.g., [35, 49, 52, 56, 59, 66, 90, 95, 105]). Variables such as total distance, Player Load, and acceleration volume were frequently associated with TRIMP, %HRmax, or HR zone-based metrics. For example, TRIMP based parameters correlated strongly with total distance (*r* = .79–0.84) and Player Load (*r* = .81–0.85) [59], while Player Load alone explained up to 87% of the variance in HR response during intermittent field hockey training [66]. Other approaches employed ratio-based indices to relate external and internal load [103, 107, 111]. These indices confirmed differences according to playing role and exposure time, with HR-derived indicators (e.g., HRmax, cardiovascular capacity) varying by position and match progression, and efficiency indexes (effindex: mean speed/%HRmax) differentiating positions and match halves. Similar comparisons of average heart rate, external intensity, and performance index were also used to examine external–internal load relationships [91].

In contrast, heart rate variability (HRV) indicators such as lnRMSSD showed trivial to small associations with external load in several studies [39, 44, 55, 67, 86]. For instance, external load decreased significantly during a two-week preseason, yet HRV remained unchanged [62], while non-significant correlations (*r* < .20) were observed between HRV and training volume or distance [74]. An exception was reported by Sánchez-Sánchez et al. [93], who found negative associations between pre-match HRV parameters (e.g., LF/HF ratio) and in-match external load metrics such as sprint distance and sprint count.

Session RPE (sRPE) consistently showed moderate to strong correlations with external load (e.g., [36, 45, 48, 56, 89, 99]). For example, a one-unit increase in RPE predicted an additional 106 m of high-speed running and 49 AU of Player Load [52], while sRPE explained up to 65% of the variance in TRIMP [106]. Data were commonly collected via smartphone applications [43, 96] or standardized post-session scales [63, 106].

Wellness measures such as the perception of fatigue, soreness, sleep quality, and stress were assessed in 24 studies (e.g., [41, 53, 74, 82, 102]). High external loads, particularly accelerations and match load, were negatively associated with subsequent wellness measures (perception of fatigue, soreness, sleep quality, and stress), especially on the first (MD + 1) and second (MD + 2) day after the match. Strong associations were observed between total distance and both muscle soreness (*r* = –.67) and perceived stress (*r* = –.70) [89]. Conversely, other studies found no significant associations between wellness ratings and load metrics [44, 50, 67, 68]. For instance, prematch wellness scores showed only trivial relationships with match load outcomes [44]. Sleep quality and perceived energy emerged as the most consistent predictors, with significant associations reported in several studies [53, 82, 109]. Notably, athletes with good sleep quality covered ~ 300 m more per training day compared with those reporting poor sleep [53].

### Relationship between subjective and objective internal load measures

Seven studies directly compared subjective (sRPE, RPE, wellness) and objective (HR, TRIMP) internal load measures. A moderate correlation (*r* = .45) between sRPE and Summated Heart Rate Zones (SHRZ) was reported [94]. Shared variances between sRPE, TRIMP, and SHRZ ranged from 14% to 38%, indicating partial overlap between perceptual and physiological load markers [96]. sRPE-TL predicted TRIMP with high accuracy (R² = 0.65), and model fit improved by an additional 11% when total distance was included [106]. Comparisons between RPE and HR-derived indices also revealed statistically significant associations [51], while perceived exertion ratings were moderately aligned with SHRZ and %HRmax metrics in elite football contexts [63]. Studies on wellness-based load perception demonstrated weak or inconsistent correlations with HR and TRIMP [50], although others noted stronger alignment between wellness indicators and HR zones [80].

### Contextual and individual modifiers

Contextual factors influenced load-response relationships across multiple studies. Changes in both external and internal load were associated with different competition phases, such as pre-season versus international tournaments [64, 108]. During high-density match periods, training loads were reduced while game loads remained high [85]. Position-specific differences in workload and physiological response indicated that player role modifies both external load exposure and internal responses (physiological and perceptual) [91, 101]. For instance, players in more physically demanding positions may accumulate greater high-intensity efforts and exhibit stronger cardiovascular responses than those in lower demanding roles, thereby influencing their subjective perception of effort.

## Discussion

The primary objective of this scoping review was to identify EMA methodologies using mHealth technologies for monitoring training load or the stress-recovery process in athletes. Specifically, the review aimed to classify the methodologies and technologies used according to individual or team sports, to describe the relationship between external parameters (e.g., distance, accelerations) and internal parameters (e.g., physiological, cardiovascular, neuromuscular, subjective), and to analyze the relationship between subjective and objective internal load measures.

The findings align in part with previous systematic reviews [29, 30]. Like those works, this review confirms moderate to very strong associations between heart rate–derived internal load (e.g., TRIMP, %HRmax) and external load variables (e.g., total distance, Player Load) across sports and contexts [42, 59, 66, 97, 102]. However, this review extends prior findings by incorporating a wider range of internal load constructs and exploring differences by sport type, competitive context, and data acquisition methodology.

An important contribution of this review is the identification of advanced analytical approaches. For instance, Campbell et al. [41] applied machine learning models to predict training load from wellness scores, although these models showed limited predictive power. In addition, Rago et al. [88] used unsupervised machine learning to reveal strong associations between internal and external load measures and to identify parameters that distinguished between player groups and temporal patterns. Others studies used canonical correlations [42], stepwise regression [78], or multilinear models [99] to explore multidimensional interactions. While still underused, these approaches point toward a more sophisticated, data-informed future in athlete monitoring.

An important distinction that emerged from this review relates to sport type and gender. Studies mainly focused on team sports (*n* = 62), especially soccer (*n* = 33), while individual sports (*n* = 9) were notably underrepresented. Given that individual athletes often follow highly personalized training and recovery patterns, this imbalance reveals a clear gap in the literature and highlights the need for future research exploring EMA-based monitoring strategies tailored to individual sport contexts. In terms of gender, 45 studies included exclusively male athletes (*n* = 1120), whereas only 21 focused on female participants (*n* = 323). This gender disparity further underscores the need to expand research efforts to better represent female athletes and their specific training and recovery profiles. Although four studies recruited mixed-gender samples, none reported separate analyses for male and female athletes in the relationship between internal and external loads. In addition, the review revealed important differences in technological accessibility. While elite-level environments frequently employed advanced systems such as Catapult, STATSports, or Polar Team Pro, these tools are often cost-prohibitive for clubs, individual athletes, or teams with limited budgets. However, several studies demonstrated that simpler, low-cost alternatives such as mobile apps or basic subjective scales can still deliver valid and useful data [43, 96]. These findings emphasize the potential of accessible monitoring solutions to extend best practices beyond high-performance environments and into more resource-limited or amateur settings, supporting more equitable athlete care and decision-making.

Regarding the relationship between external and internal load, most studies confirmed robust associations (*r* = .79–0.85). In addition to classic correlations, some adopted derived indices to evaluate internal-to-external load ratios [91, 103, 107], while others related HR-derived cardiovascular capacity to positional demands [111]. These indices offered deeper insights into athlete adaptation and performance economy. By contrast, HRV metrics such as lnRMSSD showed limited responsiveness to training load in many studies [46, 62, 74], supporting previous claims that HRV is more effective for long-term recovery monitoring than short-term load assessment. Nevertheless, recent advancements in wearable biosensing technologies [112] now enable the non-invasive and continuous monitoring of internal load parameters like lactate or glucose, via sweat. These parameters were previously restricted to invasive blood-based methods, and their integration into mHealth systems represents a promising step toward more comprehensive and real-time internal load monitoring in the field.

Subjective measures, particularly sRPE, proved reliable and were moderately to strongly correlated with external load [40, 49, 52, 93, 103]. Components of wellness like sleep quality and perceived energy were among the most predictive variables [53, 109], though others like stress or soreness yielded inconsistent results [44, 67, 68]. However, this review revealed that many studies did not report how subjective variables were collected, and several still used non-digital tools, despite the demonstrated benefits of mobile-based EMA approaches in improving data quality and ecological validity [21]. This lack of methodological transparency and technological integration has also been highlighted in practice-focused studies [113], showing that subjective measures are widely used but often inconsistently applied, with limited feedback to athletes and little standardization. Our findings complement this by showing that while subjective metrics are valuable and commonly used, their implementation often lacks the rigor and integration found in objective monitoring.

Moreover, only 7 studies compared subjective and objective internal load. These studies compared physiological metrics such as TRIMP, %HRmax, or SHRZ with perceptual indicators like sRPE or wellness scores. Results revealed a partial convergence between these two domains: although correlations were generally significant, the shared variance between sRPE and TRIMP-type models ranged from 14% to 38% [94, 96, 106]. This indicates that while sRPE captures a meaningful portion of physiological load, it also reflects additional psychological, contextual, or perceptual dimensions not accounted for by objective measures. Beyond this, sRPE has become one of the most widely adopted subjective indicators, by conveying a more global sense of effort relative to a given external load [114]. However, the substantial variance not shared with physiological markers such as TRIMP suggests that other aspects of cognitive exertion may play an important role. Incorporating such cognitive measures, particularly when collected in ecological settings through mHealth solutions, may offer a complementary perspective that is less invasive, more affordable, and broadly feasible across sporting environments. This approach aligns with the need for multimodal monitoring strategies, especially in field-based contexts where physiological data may not always be accessible. They also reinforce the notion that subjective data—far from being secondary—provide unique insights into how athletes perceive and integrate training stimuli, including stress, fatigue, and readiness perception. Subjective and objective measures should not be treated as interchangeable, but rather as synergistic components of athlete monitoring. Furthermore, these align with recent views that athlete monitoring must be framed within a complex systems perspective [20]. In this view, perception, recovery behaviors, and environmental context are not peripheral but central to understanding load adaptation. By analyzing both types of internal load and their relationship to external demands, this review contributes to a more integrated and ecologically valid model of athlete monitoring. The potential impact lies in promoting multimodal, ecologically valid approaches that incorporate not only physiological and perceptual load, but also recovery behaviors and emerging biosensing technologies. In applied practice, such frameworks can guide coaches and clinicians in tailoring interventions, preventing injury, and optimizing adaptation, while stimulating the development of future sport-specific guidelines.

This scoping review is subject to several limitations. First, although the search strategy was comprehensive, relevant studies may have been missed due to database indexing limitations, language restrictions, or the exclusion of grey literature. Second, the evidence base identified within the selected timeframe (2012–2025) was skewed toward team sports, particularly soccer, and toward elite/international male athletes. Importantly, the comparatively lower number of included studies in individual sports should be interpreted cautiously, as it may reflect the post-2012 expansion and uptake of mHealth/EMA tools in intermittent team-sport settings rather than a definitive lack of monitoring research in individual disciplines. In contrast, the under-representation of female cohorts constitutes a more substantive evidence gap and limits the generalizability of current findings. Third, the heterogeneity of methodologies, technologies, and measurement tools used across studies made direct comparisons difficult. In particular, the lack of standardization in the collection and reporting of subjective internal load measures, such as RPE or wellness scores was notable, as many studies did not specify the format or timing of data collection. Future research would benefit from the adoption of more standardised protocols. Table 3 provides an example checklist to support consistent implementation and reporting of these measures, including: (1) scale specification (2) timing of administration in relation to training or competition (3) mode of administration, (4) contextual factors, and (5) reporting procedures for data aggregation and interpretation.

**Table 3:** Checklist to ensure consistent use of subjective measures

Scale specification	CR-10, 0–100 for RPE; number and type of items for wellness questionnaires
Timing of administration	Pre-session for wellness, post-session for RPE
Mode of administration	App, online forms, verbal
Contextual factors	Individual vs. group setting, environmental conditions
Aggregation & interpretation	Derived summary scores, decision thresholds, missing-data handling, and dashboard-style visualization.

*RPE* Rating of Perceived Exertion

Moreover, there were substantial differences in the periodicity of data collection (e.g., daily, weekly, post-session) and in the characteristics of the participants, which ranged widely in age, competitive level, and sport discipline. Additionally, even within the same technology brands as Catapult, STATSports or Polar, different models and device generations were used across studies, potentially affecting the consistency and comparability of the collected data. This variability may limit the comparability and transferability of findings across contexts.

## Conclusions

This scoping review provides an updated and comprehensive overview of Ecological Momentary Assessment methodologies using mHealth technologies to monitor training load and the stress-recovery process in athletes. The findings confirm well-established relationships between external load variables and heart rate–based internal measures, while highlighting the growing role of subjective metrics such as sRPE and wellness scores. However, only seven studies directly compared subjective and objective internal load, revealing partial convergence and underscoring the need for multimodal monitoring strategies that combine both types of data to better capture athletes’ psychophysiological responses. This review thus reinforces recent perspectives that advocate for a complex systems approach, where perception, recovery behaviour, and environmental context are integral to understanding training adaptation.

Moreover, the emergence of non-invasive, continuous biosensing technologies capable of capturing internal load markers such as lactate and glucose opens new avenues for real-time, field-based monitoring that complements traditional methods. In parallel, this review identified a notable lack of attention to recovery-related behaviours, which remain insufficiently monitored despite their crucial role in performance and injury prevention. From a practical standpoint, coaches and practitioners seeking to implement EMA and mHealth-based monitoring systems should prioritize simplicity, consistency, and athlete engagement. The choice of tools ought to be guided not only by technological sophistication but also by ease of use, low burden, and compatibility with existing routines.

Future research should prioritize greater methodological transparency, particularly in the collection of subjective data, extend monitoring strategies to individual sports, and incorporate behavioural recovery markers into EMA-based systems. Doing so will bring the field closer to a truly integrated and ecologically valid model of athlete monitoring. A key agenda is the development of personalized models of monitoring, in which individual stress–recovery profiles, thresholds, and early-warning signals of maladaptation can be identified. In addition, more sophisticated quantification of recovery is needed, integrating physiological, psychological, and behavioral markers into coherent indices that can inform real-time adjustments to training prescription. Finally, advances in machine learning and AI-driven analytics offer promising opportunities to handle the complexity of multimodal data, identify non-linear interactions among training loads and performance, and generate individualized recommendations.

## Supplementary Information

Supplementary Material 1.

## Data Availability

The data supporting the findings of this study are openly available in the Catalan Open Research Area repository at [https://doi.org/10.34810/data2613](https:/doi.org/10.34810/data2613).

## References

[1] Skau S, Sundberg K, Kuhn H-G. A Proposal for a Unifying Set of Definitions of Fatigue. Front Psychol. 2021;12. 10.3389/fpsyg.2021.739764. 10.3389/fpsyg.2021.739764 PMC8548736 34721213

[2] Heidari J, Beckmann J, Bertollo M, Brink M, Kallus KW, Robazza C, et al. Multidimensional Monitoring of Recovery Status and Implications for Performance. Int J Sports Physiol Perform. 2019;14:2–8. 10.1123/ijspp.2017-0669. 29543069 10.1123/ijspp.2017-0669

[3] Kellmann M, Bertollo M, Bosquet L, Brink M, Coutts AJ, Duffield R, et al. Recovery and Performance in Sport: Consensus Statement. Int J Sports Physiol Perform. 2018;13:240–5. 10.1123/ijspp.2017-0759. 29345524 10.1123/ijspp.2017-0759

[4] Thorpe RT, Atkinson G, Drust B, Gregson W. Monitoring Fatigue Status in Elite Team-Sport Athletes: Implications for Practice. Int J Sports Physiol Perform. 2017;12. 10.1123/ijspp.2016-0434. S2-27-S2-34. 10.1123/ijspp.2016-0434 28095065

[5] McGuigan H, Hassmén P, Rosic N, Stevens CJ. Training monitoring methods used in the field by coaches and practitioners: A systematic review. Int J Sports Sci Coach. 2020;15:439–51. 10.1177/1747954120913172.

[6] Halson SL. Monitoring Training Load to Understand Fatigue in Athletes. Sports Med. 2014;44:139–47. 10.1007/s40279-014-0253-z. 10.1007/s40279-014-0253-z PMC4213373 25200666

[7] Gabbett TJ. Load Management in Basketball. In: Laver L, Kocaoglu B, Cole B, Arundale AJH, Bytomski J, Amendola A, editors. Basketball Sports Medicine and Science. Berlin, Heidelberg: Springer; 2020. pp. 815–22. 10.1007/978-3-662-61070-1_64.

[8] Impellizzeri FM, Marcora SM, Coutts AJ. Internal and External Training Load: 15 Years On. Int J Sports Physiol Perform. 2019;14:270–3. 10.1123/ijspp.2018-0935. 30614348 10.1123/ijspp.2018-0935

[9] Vanrenterghem J, Nedergaard NJ, Robinson MA, Drust B. Training Load Monitoring in Team Sports: A Novel Framework Separating Physiological and Biomechanical Load-Adaptation Pathways. Sports Med. 2017;47:2135–42. 10.1007/s40279-017-0714-2. 28283992 10.1007/s40279-017-0714-2

[10] Bourdon PC, Cardinale M, Murray A, Gastin P, Kellmann M, Varley MC, et al. Monitoring Athlete Training Loads: Consensus Statement. Int J Sports Physiol Perform. 2017;12:S2–170. 10.1123/IJSPP.2017-0208. 10.1123/IJSPP.2017-0208 28463642

[11] Torres-Ronda L, Beanland E, Whitehead S, Sweeting A, Clubb J. Tracking Systems in Team Sports: A Narrative Review of Applications of the Data and Sport Specific Analysis. Sports Med - Open. 2022;8:15. 10.1186/s40798-022-00408-z. 35076796 10.1186/s40798-022-00408-z PMC8789973

[12] Coutts AJ, Crowcroft S, Kempton T. Developing athlete monitoring systems: Theoretical basis and practical applications. Recovery and Well-being in Sport and Exercise. Routledge; 2021.

[13] Mujika I, Halson S, Burke LM, Balagué G, Farrow D. An Integrated, Multifactorial Approach to Periodization for Optimal Performance in Individual and Team Sports. 2018. 10.1123/ijspp.2018-0093 10.1123/ijspp.2018-0093 29848161

[14] Bishop PA, Jones E, Woods AK. Recovery From Training: A Brief Review: Brief Review. J Strength Cond Res. 2008;22:1015. 10.1519/JSC.0b013e31816eb518. 18438210 10.1519/JSC.0b013e31816eb518

[15] Düking P, Achtzehn S, Holmberg H-C, Sperlich B. Integrated Framework of Load Monitoring by a Combination of Smartphone Applications, Wearables and Point-of-Care Testing Provides Feedback that Allows Individual Responsive Adjustments to Activities of Daily Living. Sensors. 2018;18:1632. 10.3390/s18051632. 29783763 10.3390/s18051632 PMC5981295

[16] Weaving D, Jones B, Till K, Abt G, Beggs C. The Case for Adopting a Multivariate Approach to Optimize Training Load Quantification in Team Sports. Front Physiol. 2017;8. 10.3389/fphys.2017.01024. 10.3389/fphys.2017.01024 PMC5732925 29311959

[17] World Health Assembly 71. mHealth: use of appropriate digital technologies for public health: report by the Director-General. 2018.

[18] Shiffman S, Stone AA, Hufford MR. Ecological Momentary Assessment. Annu Rev Clin Psychol. 2008;4 Volume 4, 2008:1–32. 10.1146/annurev.clinpsy.3.022806.091415 10.1146/annurev.clinpsy.3.022806.091415 18509902

[19] West SW, Clubb J, Torres-Ronda L, Howells D, Leng E, Vescovi JD, et al. More than a Metric: How Training Load is Used in Elite Sport for Athlete Management. Int J Sports Med. 2021;42:300–6. 10.1055/a-1268-8791. 33075832 10.1055/a-1268-8791

[20] Montull L, Slapšinskaitė-Dackevičienė A, Kiely J, Hristovski R, Balagué N. Integrative Proposals of Sports Monitoring: Subjective Outperforms Objective Monitoring. Sports Med - Open. 2022;8:41. 10.1186/s40798-022-00432-z. 35348932 10.1186/s40798-022-00432-z PMC8964908

[21] Timmerman WP, Abbiss CR, Lawler NG, Stanley M, Raynor AJ. Athlete monitoring perspectives of sports coaches and support staff: A scoping review. Int J Sports Sci Coach. 2024;19:1813–32. 10.1177/17479541241247131.

[22] Fox JL, Stanton R, Sargent C, Wintour S-A, Scanlan AT. The Association Between Training Load and Performance in Team Sports: A Systematic Review. Sports Med. 2018;48:2743–74. 10.1007/s40279-018-0982-5. 30225537 10.1007/s40279-018-0982-5

[23] Petway AJ, Freitas TT, Calleja-González J, Medina Leal D, Alcaraz PE. Training load and match-play demands in basketball based on competition level: A systematic review. PLoS ONE. 2020;15:e0229212. 10.1371/journal.pone.0229212. 32134965 10.1371/journal.pone.0229212 PMC7058381

[24] Saw AE, Main LC, Gastin PB. Monitoring the athlete training response: subjective self-reported measures trump commonly used objective measures: a systematic review. Br J Sports Med. 2016;50:281–91. 10.1136/bjsports-2015-094758. 26423706 10.1136/bjsports-2015-094758 PMC4789708

[25] Teixeira JE, Forte P, Ferraz R, Leal M, Ribeiro J, Silva AJ, et al. Monitoring Accumulated Training and Match Load in Football: A Systematic Review. Int J Environ Res Public Health. 2021;18:3906. 10.3390/ijerph18083906. 33917802 10.3390/ijerph18083906 PMC8068156

[26] Adesida Y, Papi E, McGregor AH. Exploring the Role of Wearable Technology in Sport Kinematics and Kinetics: A Systematic Review. Sensors. 2019;19:1597. 10.3390/s19071597. 30987014 10.3390/s19071597 PMC6480145

[27] Cummins C, Orr R, O’Connor H, West C. Global Positioning Systems (GPS) and Microtechnology Sensors in Team Sports: A Systematic Review. Sports Med. 2013;43:1025–42. 10.1007/s40279-013-0069-2. 23812857 10.1007/s40279-013-0069-2

[28] Seshadri DR, Li RT, Voos JE, Rowbottom JR, Alfes CM, Zorman CA, et al. Wearable sensors for monitoring the internal and external workload of the athlete. Npj Digit Med. 2019;2:71. 10.1038/s41746-019-0149-2. 31372506 10.1038/s41746-019-0149-2 PMC6662809

[29] McLaren SJ, Macpherson TW, Coutts AJ, Hurst C, Spears IR, Weston M. The Relationships Between Internal and External Measures of Training Load and Intensity in Team Sports: A Meta-Analysis. Sports Med. 2018;48:641–58. 10.1007/s40279-017-0830-z. 29288436 10.1007/s40279-017-0830-z

[30] Helwig J, Diels J, Röll M, Mahler H, Gollhofer A, Roecker K, et al. Relationships between External, Wearable Sensor-Based, and Internal Parameters: A Systematic Review. Sensors. 2023;23:827. 10.3390/s23020827. 36679623 10.3390/s23020827 PMC9864675

[31] Anderson N, Robinson DG, Verhagen E, Fagher K, Edouard P, Rojas-Valverde D, et al. Under-representation of women is alive and well in sport and exercise medicine: what it looks like and what we can do about it. BMJ Open Sport Exerc Med. 2023;9. 10.1136/bmjsem-2023-001606. 10.1136/bmjsem-2023-001606 PMC10186450 37200777

[32] de Jager E, Caulfield B, Angelidi E, MacNamee Brian HS. Wearable-Derived Heart Rate Variability Across the Menstrual Cycle, Hormonal Contraceptive Use, and Reproductive Life Stages in Females: A Living Systematic Review. Sports Med. 2026. 10.1007/s40279-025-02388-y. 41545627 10.1007/s40279-025-02388-y PMC13198475

[33] Tricco AC, Lillie E, Zarin W, O’Brien KK, Colquhoun H, Levac D, et al. PRISMA Extension for Scoping Reviews (PRISMA-ScR): Checklist and Explanation. Ann Intern Med. 2018;169:467–73. 10.7326/M18-0850. 30178033 10.7326/M18-0850

[34] Guillen J, Capdevila L, Losilla J-M, Parrado E. Ecological Momentary Assessment of the stress-recovery process through technology mHealth in sports: a scoping review protocol. INPLASY - International Platform of Registered Systematic Review and Meta-analysis Protocols; 2023. 10.37766/inplasy2023.3.0089

[35] McGowan J, Sampson M, Salzwedel DM, Cogo E, Foerster V, Lefebvre C. PRESS Peer Review of Electronic Search Strategies: 2015 Guideline Statement. J Clin Epidemiol. 2016;75:40–6. 10.1016/j.jclinepi.2016.01.021. 27005575 10.1016/j.jclinepi.2016.01.021

[36] Rethlefsen ML, Kirtley S, Waffenschmidt S, Ayala AP, Moher D, Page MJ, et al. PRISMA-S: an extension to the PRISMA Statement for Reporting Literature Searches in Systematic Reviews. Syst Rev. 2021;10:39. 10.1186/s13643-020-01542-z. 33499930 10.1186/s13643-020-01542-z PMC7839230

[37] Number of European Smartphone Users Accessing News Surges. 74% Over Past Year. Comscore, Inc. https://www.comscore.com/Insights/Press-Releases/2012/3/Number-of-European-Smartphone-Users-Accessing-News-Surges-74-Percent-Over-Past-Year

[38] Fanning J, Mullen SP, McAuley E. Increasing Physical Activity With Mobile Devices: A Meta-Analysis. J Med Internet Res. 2012;14:e2171. 10.2196/jmir.2171. 10.2196/jmir.2171 PMC3514847 23171838

[39] Grant MJ, Booth A. A typology of reviews: an analysis of 14 review types and associated methodologies. Health Inf Libr J. 2009;26:91–108. 10.1111/j.1471-1842.2009.00848.x. 10.1111/j.1471-1842.2009.00848.x 19490148

[40] McKay AKA, Stellingwerff T, Smith ES, Martin DT, Mujika I, Goosey-Tolfrey VL, et al. Defining Training and Performance Caliber: A Participant Classification Framework. Int J Sports Physiol Perform. 2021;17:317–31. 10.1123/ijspp.2021-0451. 10.1123/ijspp.2021-0451 34965513

[41] Campbell PG, Stewart IB, Sirotic AC, Drovandi C, Foy BH, Minett GM. Analysing the predictive capacity and dose-response of wellness in load monitoring. J Sports Sci. 2021;39:1339–47. 10.1080/02640414.2020.1870303. 33404378 10.1080/02640414.2020.1870303

[42] Abdullahi Y, Coetzee B, van den Berg L. Relationships Between Results of an Internal and External Match Load Determining Method in Male, Singles Badminton Players. J Strength Cond Res. 2019;33:1111–8. 10.1519/JSC.0000000000002115. 28682932 10.1519/JSC.0000000000002115

[43] Askow AT, Lobato AL, Arndts DJ, Jennings W, Kreutzer A, Erickson JL, et al. Session Rating of Perceived Exertion (sRPE) Load and Training Impulse Are Strongly Correlated to GPS-Derived Measures of External Load in NCAA Division I Women’s Soccer Athletes. J Funct Morphol Kinesiol. 2021;6. 10.3390/jfmk6040090. 10.3390/jfmk6040090 PMC8628997 34842757

[44] Bellinger PM, Ferguson C, Newans T, Minahan CL. No Influence of Prematch Subjective Wellness Ratings on External Load During Elite Australian Football Match Play. Int J Sports Physiol Perform. 2020;15:801–7. 10.1123/ijspp.2019-0395. 32053792 10.1123/ijspp.2019-0395

[45] Bisschoff CA, Coetzee B, Esco MR. Relationship between heart rate, heart rate variability, heart rate recovery and global positioning system determined match characteristics of male, elite, African badminton players. Int J Perform Anal SPORT. 2016;16:881–97.

[46] Chrismas BCR, Taylor L, Thornton HR, Murray A, Stark G. External training loads and smartphone-derived heart rate variability indicate readiness to train in elite soccer. Int J Perform Anal SPORT. 2019;19:143–52. 10.1080/24748668.2019.1578097.

[47] Clemente FM. Associations between wellness and internal and external load variables in two intermittent small-sided soccer games. Physiol Behav. 2018;197:9–14. 10.1016/j.physbeh.2018.09.008. 30236526 10.1016/j.physbeh.2018.09.008

[48] Clements E, Ehrmann F, Clark A, Jones M, McCall A, Duffield R. Influence of Travel Demands and Match Load on Recovery Following Postmatch Travel in National-Team Footballers. Int J Sports Physiol Perform. 2025;1–9. 10.1123/ijspp.2024-0211. 10.1123/ijspp.2024-0211 40010360

[49] Conte D, Kamarauskas P, Ferioli D, Scanlan AT, Kamandulis S, Paulauskas H, et al. Workload and well-being across games played on consecutive days during in-season phase in basketball players. J Sports Med Phys Fit. 2021;61. 10.23736/s0022-4707.20.11396-3. 10.23736/S0022-4707.20.11396-3 33092332

[50] Conte D, Guerriero A, Lupo C, Schultz Arruda AF, Kamarauskas P. Influence of Congested Match Schedules, Pre-Match Well-Being and Level of Opponents on Match Loads during World Rugby Women’s Sevens Series. Int J Environ Res Public Health. 2021;18. 10.3390/ijerph182212132. 10.3390/ijerph182212132 PMC8623102 34831889

[51] Costa J, Figueiredo P, Nakamura F, Rago V, Rebelo A, Brito J. Intra-individual variability of sleep and nocturnal cardiac autonomic activity in elite female soccer players during an international tournament. PLoS ONE. 2019;14:e0218635. 10.1371/journal.pone.0218635. 31527865 10.1371/journal.pone.0218635 PMC6748428

[52] Croteau F, Gaudet S, Briand J, Clément J. Case study of IMU loads and self-reported fatigue monitoring of water polo goalkeepers preparing for the Olympic games. Front Sports Act Living. 2023;5:1198003–1198003. 10.3389/fspor.2023.1198003. 37255727 10.3389/fspor.2023.1198003 PMC10225700

[53] Crouch AK, Jiroutek MR, Snarr RL, Bunn JA. Relationship between pre-training wellness scores and internal and external training loads in a Division I women’s lacrosse team. J Sports Sci. 2021;39:1070–6. 10.1080/02640414.2020.1857106. 33393411 10.1080/02640414.2020.1857106

[54] Cullen BD, McCarren AL, Malone S. Ecological validity of self-reported wellness measures to assess pre-training and pre-competition preparedness within elite Gaelic football. Sport Sci Health. 2021;17:163–72.

[55] de Dios-Álvarez V, Suárez-Iglesias D, Bouzas-Rico S, Alkain P, González-Conde A, Ayán-Pérez C. Relationships between RPE-derived internal training load parameters and GPS-based external training load variables in elite young soccer players. Res Sports Med. 2023;31:58–73. 34121539 10.1080/15438627.2021.1937165

[56] Delaney JA, Duthie GM, Thornton HR, Pyne DB. Quantifying the relationship between internal and external work in team sports: development of a novel training efficiency index. Sci Med Footb. 2018;2:149–56. 10.1080/24733938.2018.1432885.

[57] Douchet T, Humbertclaude A, Cometti C, Paizis C, Babault N. Quantifying Accelerations and Decelerations in Elite Women Soccer Players during Regular In-Season Training as an Index of Training Load. Sports Basel Switz. 2021;9. 10.3390/sports9080109. 10.3390/sports9080109 PMC8402484 34437370

[58] Draper G, Wright M, Chesterton P, Atkinson G. The tracking of internal and external training loads with next-day player-reported fatigue at different times of the season in elite soccer players. Int J SPORTS Sci Coach. 2021;16:793–803. 10.1177/1747954121988960.

[59] Ellis M, Penny R, Wright B, Noon M, Myers T, Akubat I. The dose-response relationship between training-load measures and aerobic fitness in elite academy soccer players. Sci Med Footb. 2021;5:128–36. 10.1080/24733938.2020.1817536. 35077333 10.1080/24733938.2020.1817536

[60] Evans DA, Jackson DT, Kelly AL, Williams CA, McAuley ABT, Knapman H, et al. Monitoring Postmatch Fatigue During a Competitive Season in Elite Youth Soccer Players. J Athl Train. 2022;57:184–90. 10.4085/1062-6050-0245.21. 34543430 10.4085/1062-6050-0245.21 PMC8876877

[61] Fernandez D, Moya D, Cadefau JA, Carmona G, Fernández D, Moya D, et al. Integrating External and Internal Load for Monitoring Fitness and Fatigue Status in Standard Microcycles in Elite Rink Hockey. Front Physiol. 2021;12:698463. 10.3389/fphys.2021.698463. 34267678 10.3389/fphys.2021.698463 PMC8276020

[62] Fields JB, Merigan JM, Gallo S, White JB, Jones MT. External and Internal Load Measures During Preseason Training in Men Collegiate Soccer Athletes. J Strength Cond Res. 2021;35:2572–8. 10.1519/JSC.0000000000004092. 34431484 10.1519/JSC.0000000000004092

[63] Fox JL, O’Grady CJ, Scanlan AT. The Relationships Between External and Internal Workloads During Basketball Training and Games. Int J Sports Physiol Perform. 2020;15:1081–6. 10.1123/ijspp.2019-0722. 32814307 10.1123/ijspp.2019-0722

[64] Fox JL, Stanton R, Scanlan AT. A Comparison of Training and Competition Demands in Semiprofessional Male Basketball Players. Res Q Exerc Sport. 2018;89:103–11. 10.1080/02701367.2017.1410693. 29334021 10.1080/02701367.2017.1410693

[65] Gielen J, Mehuys E, Berckmans D, Meeusen R, Aerts J-M. Monitoring Internal and External Load During Volleyball Competition. Int J Sports Physiol Perform. 2022;17:640–5. 10.1123/ijspp.2021-0217. 35168198 10.1123/ijspp.2021-0217

[66] Gonzalez-Fimbres RA, Ramirez-Siqueiros MG, Reynoso-Sanchez LF, Quezada-Chacon JT, Miranda-Mendoza J, Hernandez-Cruz G. A new approach to quantify internal and external training load for intermittent sports. BIOTECNIA. 2019;21:26–34.

[67] González-García J, Romero-Moraleda B. The Impact of a Congested Competition Schedule on Load, Recovery, and Well-Being in Under-16 Female Soccer Players: A Comparison between Starters and Non-Starters during a Development Tournament. Appl Sci-BASEL. 2024;14. 10.3390/app14178066.

[68] Govus AD, Coutts A, Duffield R, Murray A, Fullagar H. Relationship Between Pretraining Subjective Wellness Measures, Player Load, and Rating-of-Perceived-Exertion Training Load in American College Football. Int J Sports Physiol Perform. 2018;13:95–101. 10.1123/ijspp.2016-0714. 28488913 10.1123/ijspp.2016-0714

[69] Grace S, Cooley A, Smith AM, Parker P, Bunn JA. Relationship Between Sleep Quality, Wellness, and Training Load in Division I Women’s Lacrosse Athletes. J Sport Behav. 2023;46:29–39.

[70] Heishman AD, Curtis MA, Saliba E, Hornett RJ, Malin SK, Weltman AL, NONINVASIVE ASSESSMENT OF INTERNAL AND EXTERNAL PLAYER LOAD: IMPLICATIONS FOR OPTIMIZING ATHLETIC PERFORMANCE. J STRENGTH Cond Res. 2018;32:1280–7. 10.1519/JSC.0000000000002413. 29373427 10.1519/JSC.0000000000002413

[71] Henderson MJ, Fransen J, McGrath JJ, Harries SK, Poulos N, Coutts AJ. Individual Factors Affecting Rugby Sevens Match Performance. Int J Sports Physiol Perform. 2019;14:620–6. 10.1123/ijspp.2018-0133. 30427236 10.1123/ijspp.2018-0133

[72] Ihsan M, Tan F, Sahrom S, Choo HC, Chia M, Aziz AR. Pre-game perceived wellness highly associates with match running performances during an international field hockey tournament. Eur J Sport Sci. 2017;17:593–602. 10.1080/17461391.2017.1301559. 28398168 10.1080/17461391.2017.1301559

[73] James C, Dhawan A, Jones T, Pok C, Yeo V, Girard O. Minimal Agreement between Internal and External Training Load Metrics across a 2-wk Training Microcycle in Elite Squash. J Sports Sci Med. 2021;20:101–9. 10.52082/jssm.2021.101. 33707993 10.52082/jssm.2021.101 PMC7919364

[74] Javaloyes A, Mateo-March M, Manresa-Rocamora A, Sanz-Quinto S, Moya-Ramón M. The Use of a Smartphone Application in Monitoring HRV during an Altitude Training Camp in Professional Female Cyclists: A Preliminary Study. Sensors. 2021;21. 10.3390/s21165497. 10.3390/s21165497 PMC8401324 34450939

[75] Kårström A, Swarén M, Björklund G. Discrepancies in internal and external training load measurements during low-intensity biathlon training. Front Sports Act Living. 2024;6:1455900. 10.3389/fspor.2024.1455900. 39398268 10.3389/fspor.2024.1455900 PMC11466799

[76] Kniubaite A, Skarbalius A, Clemente FM, Conte D. Quantification of external and internal match loads in elite female team handball. Biol SPORT. 2019;36:311–6. 10.5114/biolsport.2019.88753. 31938001 10.5114/biolsport.2019.88753 PMC6945049

[77] Lacome M, Simpson B, Broad N, Buchheit M. Monitoring Players’ Readiness Using Predicted Heart-Rate Responses to Soccer Drills. Int J Sports Physiol Perform. 2018;13:1273–80. 10.1123/ijspp.2018-0026. 29688115 10.1123/ijspp.2018-0026

[78] Lempke AFD, Hart JM, Hryvniak DJ, Rodu JS, Hertel J. Prospective running assessments among division I cross-country athletes. Phys Ther SPORT. 2022;55:37–45. 10.1016/j.ptsp.2022.02.003. 35183044 10.1016/j.ptsp.2022.02.003

[79] Long JW, Brown D, Farrell J, Gonzalez M, Cheever K. Relationship between Workload, Psychological State and Recovery in Female Soccer Athletes. Int J Sports Med. 2024;45:829–36. 10.1055/a-2304-3694. 38599613 10.1055/a-2304-3694

[80] Lopez-Laval I, Cirer-Sastre R, Sitko S, Corbi F, Vaquera A, Calleja-Gonzalez J, Relationship, between methodologies for load control in professional basketball. Rev Int Med Cienc Act Fis Deporte. 2022;22:649–61. 10.15366/rimcafd2022.87.014.

[81] Mara JK, Thompson KG, Pumpa KL, Ball NB. Periodization and Physical Performance in Elite Female Soccer Players. Int J Sports Physiol Perform. 2015;10:664–9. 25611789 10.1123/ijspp.2014-0345

[82] Matos S, Clemente FM, Brandão A, Pereira J, Rosemann T, Nikolaidis PT, et al. Training Load, Aerobic Capacity and Their Relationship With Wellness Status in Recreational Trail Runners. Front Physiol. 2019;10:1189. 10.3389/fphys.2019.01189. 31607945 10.3389/fphys.2019.01189 PMC6755333

[83] Peterson KD, Quiggle GT. Tensiomyographical responses to accelerometer loads in female collegiate basketball players. J Sports Sci. 2017;35:2334–41. 10.1080/02640414.2016.1266378. 27937967 10.1080/02640414.2016.1266378

[84] Pexa BS, Johnston CJ, Taylor JB, Ford KR. Training Load and Current Soreness Predict Future Delayed Onset Muscle Soreness in Collegiate Female Soccer Athletes. Int J Sports Phys Ther. 2023;18:38–49. 38050552 10.26603/001c.89890 PMC10693489

[85] Power CJ, Fox JL, Teramoto M, Dalbo VJ, Scanlan AT. Training and Game Loads Across Noncongested and Congested Weekly Microcycles During the Regular Season in a Semiprofessional Women’s Basketball Team. Int J Sports Physiol Perform. 2024;19:1455–66. 10.1123/ijspp.2023-0448. 39374918 10.1123/ijspp.2023-0448

[86] Rabbani A, Kargarfard M, Castagna C, Clemente FM, Twist C. Associations Between Selected Training-Stress Measures and Fitness Changes in Male Soccer Players. Int J Sports Physiol Perform. 2019;14:1050–7. 10.1123/ijspp.2018-0462. 30676148 10.1123/ijspp.2018-0462

[87] Rago V, Muschinsky A, Deylami K, Vigh-Larsen JF, Mohr M. Game Demands of a Professional Ice Hockey Team with Special Emphasis on Fatigue Development and Playing Position. J Hum Kinet. 2022;84:195–205. 10.2478/hukin-2022-000078. 36457463 10.2478/hukin-2022-000078 PMC9679183

[88] Rago V, Fernandes T, Mohr M. Identifying Key Training Load and Intensity Indicators in Ice Hockey Using Unsupervised Machine Learning. Res Q Exerc Sport. 2025;96:21–33. 10.1080/02701367.2024.2360162. 38959981 10.1080/02701367.2024.2360162

[89] Rahilly DO, Whelan N, Moane S, The effects of gps metrics, training and competition load on recovery in elite male gaelic football players. J Aust Strength Cond. 2023;31:16–22.

[90] Reina M, Mancha-Triguero D, García-Santos D, García-Rubio J, Ibáñez SJ. [Comparison of three methods of quantifying the training load in basketball]. RICYDE Rev Int Cienc Deporte. 2019;15:368–82. 10.5232/ricyde2019.05805. Comparación de tres métodos de cuantificación de la carga de entrenamiento en baloncesto.

[91] Reinhardt L, Schulze S, Schwesig R, Kurz E. Physical Match Performance in Sub-elite Soccer Players - Introduction of a new Index. Int J Sports Med. 2020;41:858–66. 10.1055/a-1165-1950. 32629505 10.1055/a-1165-1950

[92] Renaghan E, Wittels HL, Wittels SH, Wishon MJ, Hecocks D, Wittels ED, et al. Internal or External Training Load Metrics: Which Is Best for Tracking Autonomic Nervous System Recovery and Function in Collegiate American Football? J Funct Morphol Amp Kinesiol. 2024;9:5–null. 10.3390/jfmk9010005 PMC10971617 38535414

[93] Sánchez-Sánchez J, Botella J, Felipe Hernández JL, León M, Paredes-Hernández V, Colino E, et al. Heart Rate Variability and Physical Demands of In-Season Youth Elite Soccer Players. Int J Environ Res Public Health. 2021;18. 10.3390/ijerph18041391. 10.3390/ijerph18041391 PMC7913319 33546227

[94] Sansone P, Ceravolo A, Tessitore A, External. Internal, Perceived Training Loads and Their Relationships in Youth Basketball Players Across Different Positions. Int J Sports Physiol Perform. 2022;17:249–55. 10.1123/ijspp.2020-0962. 34583325 10.1123/ijspp.2020-0962

[95] Sansone P, Tessitore A, Makivic B, Ferioli D, Conte D. The Relationships Between Training-Load Models in 3 x 3 Basketball Games. Int J SPORTS Physiol Perform. 2025. 10.1123/ijspp.2024-0452. 40073870 10.1123/ijspp.2024-0452

[96] Scanlan AT, Wen N, Tucker PS, Dalbo VJ. The relationships between internal and external training load models during basketball training. J Strength Cond Res. 2014;28:2397–405. 10.1519/JSC.0000000000000458. 24662233 10.1519/JSC.0000000000000458

[97] Scott BR, Lockie RG, Knight TJ, Clark AC, Janse de Jonge XAK. A comparison of methods to quantify the in-season training load of professional soccer players. Int J Sports Physiol Perform. 2013;8:195–202. 10.1123/ijspp.8.2.195. 23428492 10.1123/ijspp.8.2.195

[98] Scott D, Lovell R. Individualisation of speed thresholds does not enhance the dose-response determination in football training. J Sports Sci. 2018;36:1523–32. 10.1080/02640414.2017.1398894. 29099673 10.1080/02640414.2017.1398894

[99] Silva B, Cruz G, Rocha-Rodrigues S, Clemente FM. Monitoring physical performance and training load in young surf athletes. J Hum SPORT Exerc. 2021;16:261–72. 10.14198/jhse.2021.162.03.

[100] Silva P, Santos ED, Grishin M, Rocha JM. Validity of Heart Rate-Based Indices to Measure Training Load and Intensity in Elite Football Players. J Strength Cond Res. 2018;32:2340–7. 10.1519/jsc.0000000000002057. 28614162 10.1519/JSC.0000000000002057

[101] Simpson MJ, Jenkins DG, Scanlan AT, Kelly VG. Relationships Between External- and Internal-Workload Variables in an Elite Female Netball Team and Between Playing Positions. Int J SPORTS Physiol Perform. 2020;15:841–6. 10.1123/ijspp.2019-0619. 32163926 10.1123/ijspp.2019-0619

[102] Sobolewski EJ. The Relationships between Internal and External Load Measures for Division I College Football Practice. SPORTS. 2020;8. 10.3390/sports8120165. 10.3390/sports8120165 PMC7765175 33333759

[103] Suarez-Arrones L, Torreño N, Requena B, De Sáez E, Casamichana D, Barbero-Alvarez JC, et al. Match-play activity profile in professional soccer players during official games and the relationship between external and internal load. J Sports Med Phys Fit. 2015;55:1417–22. 25289717

[104] Teixeira JE, Forte P, Ferraz R, Branquinho L, Morgans R, Silva AJ, et al. Resultant equations for training load monitoring during a standard microcycle in sub-elite youth football: a principal components approach. PeerJ. 2023;11:e15806–15806. 10.7717/peerj.15806. 37554335 10.7717/peerj.15806 PMC10405799

[105] Tendero-Ortiz E, Johnson MJ, Horsfall CM, Vondrasek JD, Grosicki GJ, Riemann BL, et al. Tournament Recovery Profiles and Physical Demands in a Collegiate Women’s Tennis Team. J Strength Cond Res. 2024;38:1786–92. 10.1519/JSC.0000000000004876. 39074238 10.1519/JSC.0000000000004876

[106] Tometz MJ, Jevas SA, Esposito PM, Annaccone AR. Validation of Internal and External Load Metrics in NCAA D1 Women’s Beach Volleyball. J STRENGTH Cond Res. 2022;36:2223–9. 10.1519/JSC.0000000000003963. 35916748 10.1519/JSC.0000000000003963

[107] Torreno N, Munguia-Izquierdo D, Coutts A, de Villarreal ES, Asian-Clemente J, Suarez-Arrones L, et al. Relationship Between External and Internal Loads of Professional Soccer Players During Full Matches in Official Games Using Global Positioning Systems and Heart-Rate Technology. Int J Sports Physiol Perform. 2016;11:940–6. 10.1123/ijspp.2015-0252. 26816391 10.1123/ijspp.2015-0252

[108] Villaseca-Vicuña R, Perez-Contreras J, Zabaloy S, Merino-Muñoz P, Valenzuela L, Burboa J, et al. Comparison of Match Load and Wellness between Friendly and World Cup Matches in Elite Female Soccer Players. Appl Sci-BASEL. 2023;13. 10.3390/app13031612.

[109] Wellman AD, Coad SC, Flynn PJ, Siam TK, McLellan CP, Perceived wellness associated with practice, and competition in national collegiate athletic association division i football players. J Strength Cond Res. 2019;33:112–24. 10.1519/JSC.0000000000002169. 28820856 10.1519/JSC.0000000000002169

[110] Wiig H, Andersen TE, Luteberget LS, Spencer M. Individual Response to External Training Load in Elite Football Players. Int J Sports Physiol Perform. 2020;15:696–704. 10.1123/ijspp.2019-0453. 32698124 10.1123/ijspp.2019-0453

[111] Zafar A, Guay S, Vinet S, Pilon F, Martens G, Prince F, et al. Heart Rate Dynamics and Quantifying Physical Fatigue in Canadian Football. Appl Sci-BASEL. 2024;14. 10.3390/app14125340.

[112] Rabost-Garcia G, Colmena V, Aguilar-Torán J, Vieyra Galí J, Punter-Villagrasa J, Casals-Terré J, et al. Non-Invasive Multiparametric Approach To Determine Sweat–Blood Lactate Bioequivalence. ACS Sens. 2023;8:1536–41. 10.1021/acssensors.2c02614. 37029741 10.1021/acssensors.2c02614 PMC10152482

[113] Neupert E, Holder T, Gupta L, Jobson SA. More than metrics: The role of socio-environmental factors in determining the success of athlete monitoring. J Sports Sci. 2024;42:323–32. 10.1080/02640414.2024.2330178. 38493350 10.1080/02640414.2024.2330178

[114] Haddad M, Stylianides G, Djaoui L, Dellal A, Chamari K. Session-RPE Method for Training Load Monitoring: Validity, Ecological Usefulness, and Influencing Factors. Front Neurosci. 2017;11. 10.3389/fnins.2017.00612. 10.3389/fnins.2017.00612 PMC5673663 29163016

